# Development of the paediatric society of the African league against rheumatism (PAFLAR) JIA registry and clinical profile of JIA in Africa from the PAFLAR JIA registry

**DOI:** 10.1186/s12969-024-01000-3

**Published:** 2024-07-22

**Authors:** Angela Nyangore Migowa, Wafa Hamdi, Soad Hashad, Hala Etayari, Awatif Abushhaiwia, Hanene Ferjani, Dorra Ben Nessib, Lobna Kharrat, Alia Fazaa, Lawrence Owino, Ayodele Faleye, Sheila Agyeiwaa Owusu, Doaa Mosad Mosa, Mervat Eissa, Samah Ismail Nasef, Gehad Gamal Elsehrawy, Rachel Odhiambo, James Orwa, Mohammed Hassan Abu-Zaid

**Affiliations:** 1 Department of Paediatrics, Aga Khan University Medical College East Africa Nairobi, P.O Box 30270, Nairobi, 00100 Kenya; 2 Research Unit, Aga Khan University Medical College East Africa Nairobi, Nairobi, Kenya; 3 Department of Population Health, Aga Khan University Medical College East Africa Nairobi, Nairobi, Kenya; 4 Department of Rheumatology, Kassab Institute - Tunis El Manar University - Faculty of Medicine of Tunis, Tunis, Tunisia; 5 Tripoli Children’s Hospital, University of Tripoli, Tripoli, Libya; 6 Department of Rheumatology, Mongi Slim Hospital - Tunis El Manar University - Faculty of Medicine of Tunis, Tunis, Tunisia; 7 Rheumatology Department, Kassab Institute of Orthopedics, Faculty of Medicine of Tunis, Tunis, Tunisia; 8 Department of Paediatrics, Faculty of Health Sciences Nairobi, University of Nairobi, Nairobi, Kenya; 9 Department of Paediatrics, Lagos State University Teaching Hospital Lagos, Lagos, Nigeria; 10 Department of Paediatrics and Child Health, University for Development Studies, Tamale, Ghana; 11 Department of Rheumatology and Rehabilitation, Faculty of Medicine, Mansoura University Hospital, Mansoura, Egypt; 12 Department of Rheumatology, Cairo University, Cairo, Egypt; 13 Rheumatology Department, Suez Canal University, Ismailia, Egypt; 14 Faculty of medicine Tanta, Tanta University, Tanta, Egypt

**Keywords:** Juvenile idiopathic arthritis, Paediatric rheumatology, Africa, Global Health

## Abstract

**Background:**

The spectrum of Juvenile Idiopathic Arthritis (JIA) in Africa is still largely unknown. We thus set out to illustrate how we set up the PAFLAR JIA registry and describe the clinical profile of Juvenile Idiopathic Arthritis across various regions in Africa.

**Methods:**

We carried out a retrospective observational cohort study where collaborators were trained on use of the existing PAFLAR REDCAP database to enter data for the JIA patients currently under their care capturing their epidemiological data, clinical features, laboratory investigations, diagnosis and therapy at initial diagnosis. Descriptive statistics including means, standard deviations, medians, interquartile ranges (IQR) for continuous variables and proportions for categorical variables were calculated as appropriate. Tests for difference between groups were performed between categorical variables using Pearson’s chi-square or Fisher’s exact tests. All analyses were performed using SPSS version 22 software.

**Results:**

We enrolled 302 patients, 58.6% (177 of 302) of whom were female. The median age of disease onset was 7 years (range 3–11 years) and the median age at diagnosis was 8.5 years (range 5–12 years). The median duration delay in diagnosis was 6 months (range 1-20.8 months). The JIA categories included Systemic JIA 18.9% (57), Oligoarticular JIA 19.2% (83), Polyarticular RF + ve 5% (15), Polyarticular RF-ve 17.9% (54), Enthesitis Related Arthritis (ERA) 18.2% (55), Psoriatic Arthritis 7% (21) and undifferentiated JIA 5.6% (17). As regards treatment the commonest therapies were NSAID therapy at 31.1%, synthetic DMARDs at 18.1%, synthetic DMARDs combined with NSAIDs at 17.5% and steroid therapy at 9.6%. Biological DMARDs accounted for 2.3% of therapies offered to our patients at diagnosis. The average JADAS score was 10.3 (range 4.8–18.2) and the average CHAQ score was 1.3 (range 0.7-2.0).

**Conclusion:**

Our study highlights strategies involved in setting up a Pan-African paediatric rheumatology registry that embraces our broad diversity and the vast spectrum of JIA in Africa while comparing the various therapies available to our patients. The PAFLAR JIA registry strives to ensure a comprehensive representation of the diverse healthcare landscapes within the continent. Further longitudinal observation studies are required to ascertain the long-term outcomes of our patients and ultimately help inform policy to create a more favorable health ecosystem to support the healthcare needs of JIA patients in Africa.

**Supplementary Information:**

The online version contains supplementary material available at 10.1186/s12969-024-01000-3.

## Introduction

Pediatric rheumatic diseases pose a significant burden of morbidity and mortality on children, their families and the society [1]. Furthermore, these diseases lead to physical disability, reduced overall quality of life and result in substantial direct and indirect financial expenses [2–4]. Assessing the burden and clinical features of these pediatric rheumatic diseases is a crucial first step toward improving healthcare accessibility and effectively leveraging existing healthcare systems for the benefit of affected patients [2–4].This effort aligns with the overarching goals set in motion during the World Health Organization (WHO) Bone and Joint decade (2000–2010) which proposed increased dedication and concerted efforts to amplify global awareness and understanding of the implications of musculoskeletal diseases [5]. Concurrently, there is an urgent need, especially in underserved regions like Africa, to initiate strategic measures and actions aimed at improving the overall quality of life for children enduring challenges of these chronic, persistent, and potentially disabling diseases throughout their lives [2–4].

Global advocacy for pediatric health aims to decrease infant and childhood mortality, with 75% of cause-specific mortality in children aged 5–14 years attributed to infectious diseases and trauma [6]. In the same age group, musculoskeletal diseases contribute to 0.1% of all-cause mortality [6]. Consequently, musculoskeletal diseases have not been a health priority in Africa [7, 8]. Prevailing medical efforts, particularly in low-income settings predominantly target preventable diseases such as acute respiratory infections, malaria, measles, diarrhea, HIV, malnutrition, and trauma. However, with the improved management of preventable diseases, a shift in health priorities toward chronic conditions, including rheumatic diseases, is anticipated [7, 8]. Hence, the significance of raising awareness about rheumatic diseases cannot be overstated in Africa [9]. By comprehensively understanding the intricacies of these diseases, we can pave the way for targeted interventions, resource allocation, and healthcare strategies that are tailored to the unique needs of pediatric rheumatology patients. This holistic approach not only facilitates improved patient outcomes but also contributes to the optimization of healthcare delivery systems [10–12].

The decision to prioritize JIA was driven by the significant impact it has on the lives of children and adolescents, influencing not only their physical health but also their overall well-being [13–15]. Additionally, the prevalence of JIA, combined with the distinct challenges it presents in the domains of diagnosis, treatment, and management, played a pivotal role in shaping our focus [13–15].

The PAFLAR JIA registry presents a unique opportunity to spearhead a systematic and organized approach to collect crucial clinical data across Africa. This data will not only provide insights into current clinical situations but will also set the basis for conducting additional research projects aimed at enhancing pediatric rheumatology healthcare in Africa. The establishment of a pediatric rheumatology registry for the African continent holds the potential to comprehensively delineate the natural progression of pediatric rheumatic diseases among patients across Africa. Furthermore, it facilitates the assessment of the clinical effectiveness and safety of various therapeutic interventions under diverse healthcare systems and specific epidemiological, infectious, and environmental settings. This contribution aids in the overall enhancement of pediatric rheumatology care in Africa. Therefore, our objective was to establish our Pan-African PAFLAR JIA registry and determine the baseline JIA patient characteristics and clinical features at the time of diagnosis across various regions in Africa.

## Methods

We outline the development of the PAFLAR JIA registry and describe retrospectively the cohort from August 2022 to December 2023.

### Study design

This was a retrospective observational cohort study where charts were reviewed from August 2022 to December 2023 to ascertain the clinical features and characteristics of JIA patients at diagnosis in various collaborative centres across Africa.

The PAFLAR Research Working Group was initiated following a call to action during the first PAFLAR virtual congress held on July 28–30, 2021. Subsequently, the group was formally established after a social media campaign among members requesting for collaboration of interested parties. Its inaugural meeting took place on November 25th, 2021. During this meeting, the vision and mission of the working group was highlighted to help foster a unified and collective working culture. Thereafter, ground rules and terms of reference for the PAFLAR Research Working Group were established and policies to guide our operations were formulated. The group formed four sub-committees i.e. scientific, ethics, investigator coordination, data management and manuscript through voluntary participation by members. Co-ordinators were elected for each sub-committee to oversee implementation of various tasks. Recruitment of interested collaborators was done via social media campaign conducted through the PAFLAR social media networks to identify interested investigators across the continent. By October 2023, the research working group comprised 42 members, with 21 expressing interest as potential investigators. Eight members successfully completed the ethical approval process in their respective centers according to PAFLAR scientific and ethical committee requirements, enabling them to include patients into the database.

### Registry site and location

The PAFLAR registry is hosted on a cloud server owned by PAFLAR. Data is captured using the PAFLAR REDCap (Research Electronic Data Capture) system—a secure web application designed for constructing and managing online surveys and databases. The choice of REDCap was made based on its distinctive features, including online or offline project design, accessibility, flexibility, multi-site access, autonomous utilization, full access to audit trails, automated export procedures, regulatory compliance, and availability in multiple languages. Data will remain active in the registry until voluntary withdrawal or the cessation of site investigators and/or patients. The enrollment process for the registry is continuous and ongoing. All registry documentation and paperwork are securely stored in a password-protected database created by the PAFLAR research team, with exclusive access granted to the PAFLAR research team and investigators. Registry records are retained for a period of 10 years after subject withdrawal or completion of the registry. Following this timeframe, all individual patient information will be appropriately discarded in accordance with the PAFLAR data policy.

### PAFLAR JIA registry design

The PAFLAR JIA registry protocol was drafted by the primary investigators and thereafter reviewed and ratified by the PAFLAR scientific and ethics subcommittee. The protocol and data collection tool was then translated into French to cater for francophone countries. The approved protocol was submitted to the local ethics committee of the various participating centres to obtain local ethical approval and thereafter submitted for verification to our PAFLAR ethics committee prior to authorization been granted to input data into REDCAP. Data collected were pseud-anonymized. Only the principal investigator of each center had access to the full identity of the patient.

After obtaining ethical approval, the principal investigator of each centre was then trained by the data registry officer on how to enter the baseline clinical data for the JIA patients they had under their follow up. After successful completion of the training, they were assigned unique user names and passwords to access the REDCap database to begin data entry. To ensure completeness and accuracy, online monitoring of the data entered and regular data quality checks were conducted by an independent program manager. Any identified errors or inconsistencies were promptly rectified based on the findings from these monitoring activities.

### Inclusion criteria for patients

Individuals aged 0–18 years diagnosed with Juvenile Idiopathic Arthritis (JIA) by a physician based on ILAR criteria were included in the study.

### Inclusion criteria for a center

Centers were included if they had a rheumatologist available for patient follow up.

### Exclusion criteria for patients

Medical records of patients were excluded if they had other connective tissue diseases with musculoskeletal involvement such as vasculitis and systemic lupus erythematosus among others.

### Exclusion criteria for a center

Centers were excluded if they were unable to demonstrate institutional and ethical approval or lacked the availability of a rheumatologist for follow-up and management.

### Retrospective cohort data

All registry data entries were extracted from medical records of pediatric rheumatology clinic visits using a standardized template. Initial medical history, baseline demographics, encompassing age, gender, race/ethnicity were derived from clinical records of patients with JIA. Physical examination data, including vital signs, height, weight, body mass index (BMI), musculoskeletal and systemic symptoms, were extracted from medical records. In order to monitor progression toward treatment goals of remission or minimally active disease, the Juvenile Arthritis Disease Activity Score (JADAS-10) was calculated. Medications administered at diagnosis were documented in a standard study tool. Results of laboratory and radiological investigations were obtained from clinical records, with no additional tests conducted unless clinically indicated by the patient’s condition. The scoring of the Child Health Assessment Questionnaire (CHAQ) to assess functionality was completed by study collaborators.

### Statistical analysis

Descriptive statistics including means, standard deviations, medians, interquartile ranges (IQR) for continuous variables and proportions for categorical variables were calculated as appropriate. Tests for difference between groups were performed between categorical variables using Pearson’s chi-square or Fisher’s exact tests. Statistical comparisons for continuous measures were conducted using Wilcoxon rank sum, Kruskal-Wallis, analysis of variance (ANOVA), or t tests depending on normality and the number of groups being compared. P-value significance level was set at 0.05. All analyses were performed using SPSS version 22 software.

### Clinical profile of PAFLAR JIA registry cohort

**Fig. 1:**
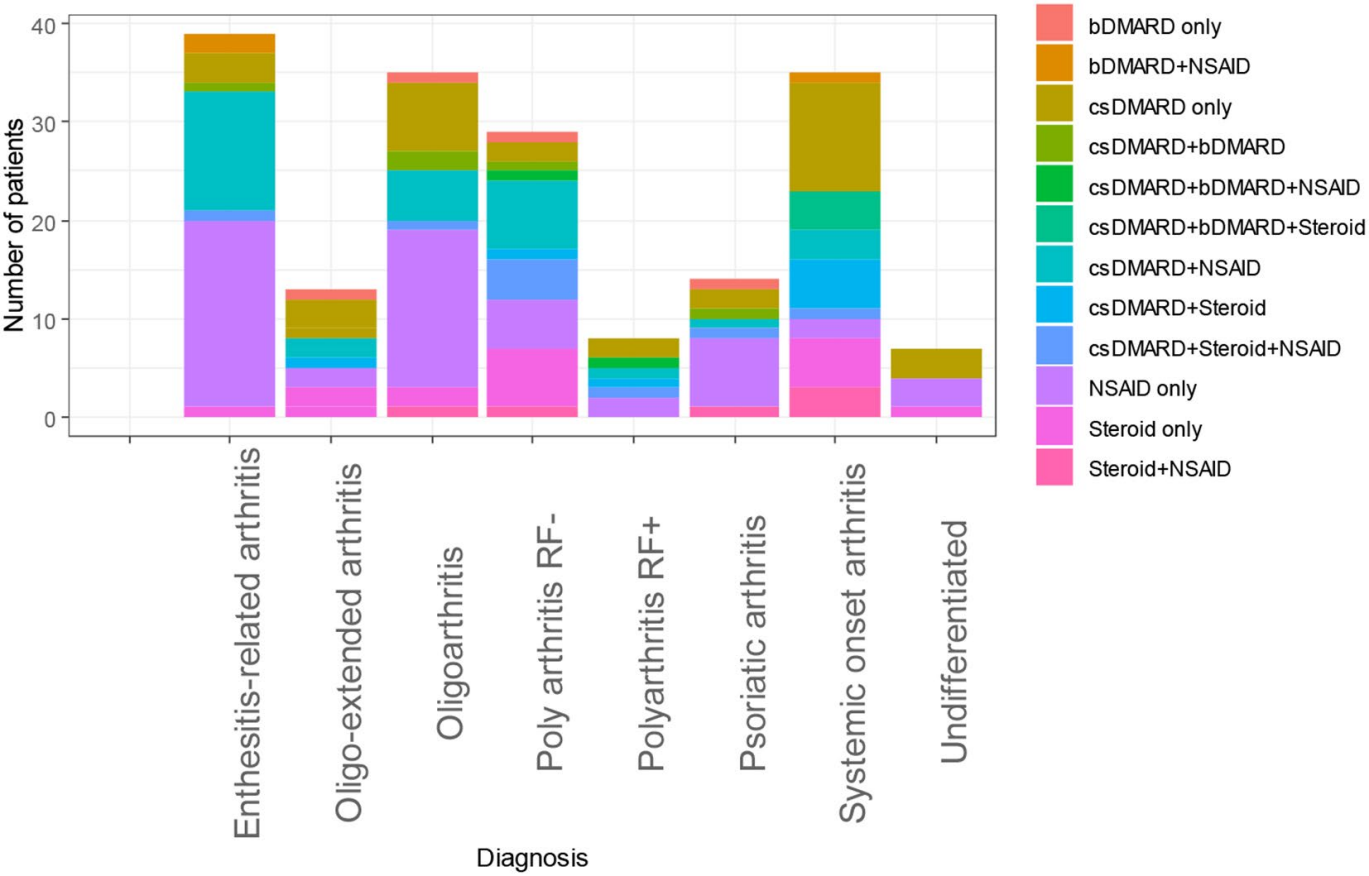
Choice of therapy for JIA Categories. RF-Rheumatoid factor. csDMARD-Synthetic Disease Modifying Anti-Rheumatic Drug. bDMARD-Biologic Disease Modifying Anti-Rheumatic Drug. NSAID-Non-Steroidal Anti-Inflammatory Drug

A total of 302 patients with JIA were enrolled into the registry. Our data highlights patients diagnosed over a duration of 23 years with earliest entry being a patient diagnosed on 15th April 2000 in Tunisia to the last being diagnosed on 5th December 2023 in Nigeria. Among the participants, 58.3% (177) were female, 23.3% (61) were of Black African descent, and 73.1% (233) were of Arab African descent. The median age of disease onset was 7 years (range 3–11 years) and the median age at diagnosis was 8.5 years (range 5–12 years). The average delay in diagnosis was 6 months (range 1-20.8 months). The distribution of ILAR categories was as follows: 27.4% (83) oligoarthritis (OligoJIA), 17.9% (54) RF-polyarticular JIA (RF-pJIA), 5% (15) RF + polyarticular JIA (RF + pJIA), 7% (16) PsJIA, 18.2% (55) ERA, 18.9% (57) systemic JIA (SJIA), and 5.6% (17) undifferentiated arthritis. The commonest presenting complaint was tender joint noted in 80.9% of the patients and the commonest extra-articular manifestations were fever (28.1%) and chronic uveitis (24.2%). At the time of inclusion, the average patient age was 7.0 ± 4.0 years, while the age at diagnosis was 8.5 ± 3.5 years. At inclusion, 80.9% (244) of patients presented with tender joints, 68.7% (207) had joint effusion and 58.4% (176) had joint deformities with limited range of motion related to their rheumatic disease. The mean JADAS 10 was 10.3 ± 6.7, and the mean CHAQ was 1.3 ± 0.65. The therapies offered to patients at diagnosis included NSAID 31.1% (55), cs DMARD 18.1% (18), cs DMARD and NSAID 17.5% (19). Biological DMARDs accounted for 2.3% (4) of therapies offered. Figure 1 illustrates the choice of therapy as per JIA category.

As regards the different categories of JIA, polyarticular RF + ve (58.3%) and undifferentiated JIA (53.8%) were commonest among the Black Africans while psoriatic arthritis (100%), Oligoarticular JIA (95.4%) and systemic JIA were commonest among the Arab Africans. The overall use of intra-articular steroids was low at 4.8% mainly among patients with ERA and Oligo JIA. The biological therapies that were available to patients during the period of the study were etanercept, adalimumab and tocilizumab. One in every 5 (22%) of the patients in our database was ANA (antinuclear antibody) positive. However, 19.7% (58) of our patients did not have the ANA test done. Rheumatoid factor was positive in 8% (20) of our patients and anti-cyclic citrullinated peptide was positive in 4.2% of our patients. 5% (14) of our patients were positive for HLA B27. The clinical-epidemiological features as per the different countries is illustrated in Table 1.

**Table 1:** Baseline clinical characteristics of Juvenile Idiopathic Arthritis (JIA) patients enrolled in PAFLAR registry from 15^TH^ April 2000–5th December 2023

**Characteristic**	**Overall**, *N* = 302^1^	Country				
		**Egypt**, *N* = 22^2^	**Kenya**, *N* = 56^2^	**Libya**, *N* = 151^2^	**Nigeria**, *N* = 7^2^	**Tunisia**, *N* = 66^2^
**Gender, n (%)**						
Female	177 (58.6)	15 (68.2)	32 (57.1)	95 (62.9)	3 (42.9)	32 (48.5)
**Ethnicity, n (%)**						
Arab African	233 (77.9)	21 (100.0)	0 (0.0)	147 (98.7)	0 (0.0)	65 (98.5)
White	4 (1.3)	0 (0.0)	4 (7.1)	0 (0.0)	0 (0.0)	0 (0.0)
Black African	61 (20.4)	0 (0.0)	52 (92.9)	1 (0.7)	7 (100.0)	1 (1.5)
Mixed Race	1 (0.3)	0 (0.0)	0 (0.0)	1 (0.7)	0 (0.0)	0 (0.0)
**Age in years, Median (IQR)**	14.0 (9.0–18.0)	9.5 (7.2–12.8)	12.0 (9.0–14.0)	16.0 (11.0–19.0)	15.0 (10.0–17.0)	13.0 (10.0–17.0)
**Age at onset (years), Median (IQR)**	7.0 (3.0–11.0)	5.0 (4.0–7.0)	7.5 (4.0–11.0)	6.0 (3.0–10.0)	10.0 (6.5–11.5)	9.0 (5.0–12.0)
**Age at diagnosis (years), Median (IQR)**	8.5 (5.0–12.0)	5.0 (4.0–7.0)	9.0 (6.0–12.5)	8.0 (5.0–11.0)	12.0 (9.5–16.0)	10.0 (6.0–14.0)
**Time to diagnosis (Months), Median (IQR)**	6.0 (1.0–20.8)	0.5 (0.0–3.8)	11.0 (1.3–33.5)	6.0 (1.0–15.8)	21.0 (9.0–30.0)	9.0 (1.0–30.0)
**JIA Categories, n (%)** **Oligoarthritis**	58 (19.2)	6 (27.3)	2 (3.6)	31 (20.5)	1 (14.3)	18 (27.3)
** Oligo-persistent arthritis**	22 (7.3)	1 (4.5)	10 (17.9)	10 (6.6)	0 (0.0)	1 (1.5)
** Oligo-extended arthritis**	6 (2.0)	0 (0.0)	0 (0.0)	5 (3.3)	0 (0.0)	1 (1.5)
**Polyarthritis RF-**	54 (17.9)	0 (0.0)	11 (19.6)	34 (22.5)	3 (42.9)	6 (9.1)
**Enthesitis-related arthritis**	55 (18.2)	0 (0.0)	8 (14.3)	20 (13.2)	0 (0.0)	27 (40.9)
**Psoriatic arthritis**	21 (7.0)	1 (4.5)	0 (0.0)	16 (10.6)	0 (0.0)	4 (6.1)
**Systemic onset arthritis**	57 (18.9)	14 (63.6)	11 (19.6)	26 (17.2)	2 (28.6)	4 (6.1)
**Polyarthritis RF+**	15 (5.0)	0 (0.0)	6 (10.7)	6 (4.0)	1 (14.3)	2 (3.0)
**Undifferentiated**	17 (5.6)	0 (0.0)	8 (14.3)	5 (3.3)	0 (0.0)	4 (6.1)
**Clinical Features, n (%)** ** Fever**	85 (28.1)	14 (63.6)	23 (41.1)	36 (23.8)	2 (28.6)	10 (15.2)
** Hepatomegaly**	11 (3.6)	4 (18.2)	3 (5.4)	3 (2.0)	1 (14.3)	0 (0.0)
** Rash**	47 (15.6)	8 (36.4)	6 (10.7)	30 (19.9)	0 (0.0)	3 (4.5)
** Splenomegaly**	5 (1.7)	3 (13.6)	0 (0.0)	1 (0.7)	0 (0.0)	1 (1.5)
** Anemia**	29 (9.6)	9 (40.9)	2 (3.6)	8 (5.3)	1 (14.3)	9 (13.6)
** Lymphadenopathy**	24 (7.9)	7 (31.8)	3 (5.4)	11 (7.3)	1 (14.3)	2 (3.0)
** Serositis**	5 (1.7)	5 (22.7)	0 (0.0)	0 (0.0)	0 (0.0)	0 (0.0)
** Psoriasis**	5 (1.7)	1 (4.5)	0 (0.0)	1 (0.7)	0 (0.0)	3 (4.5)
** Nail pits**	2 (0.7)	0 (0.0)	0 (0.0)	2 (1.3)	0 (0.0)	0 (0.0)
** Other**	22 (7.3)	0 (0.0)	10 (17.9)	11 (7.3)	0 (0.0)	1 (1.5)
**Physical exam findings, n (%)**						
** Tender joint**	241 (80.9)	22 (100.0)	39 (70.9)	118 (79.2)	7 (100.0)	55 (84.6)
** Swollen joint**	204 (68.7)	21 (95.5)	31 (56.4)	111 (74.5)	6 (85.7)	35 (54.7)
** Enthesitis**	35 (13.1)	1 (6.2)	1 (2.3)	19 (13.3)	0 (0.0)	14 (24.1)
** Joint with limited range of motion**	174 (58.4)	8 (36.4)	33 (60.0)	101 (67.8)	6 (85.7)	26 (40.0)
** Dactylitis**	9 (4.4)	1 (7.1)	2 (25.0)	3 (2.8)	3 (42.9)	0 (0.0)
** Uveitis**						
Acute	4 (6.5)	0 (0.0)	2 (66.7)	1 (6.7)	0 (0.0)	1 (2.6)
Chronic	15 (24.2)	1 (33.3)	1 (33.3)	9 (60.0)	0 (0.0)	4 (10.5)
None	43 (69.4)	2 (66.7)	0 (0.0)	5 (33.3)	3 (100.0)	33 (86.8)
**Baseline Investigations** **ESR** (*n* = 246), Median (IQR)	35.0 (15.0–60.0)	40.0 (30.0–60.0)	35.0 (20.0–64.0)	35.0 (14.0–59.0)	99.0 (41.5–122.0)	25.0 (13.0–54.8)
**CRP** (*n* = 169), Median (IQR)	13.0 (3.0–52.4)	12.0 (10.0–24.0)	22.0 (8.1–76.0)	15.5 (2.0–62.0)	232.2 (200.0–272.4)	5.9 (2.8–38.3)
**Hb levels** (*n* = 257), Median (IQR)	11.2 (10.0–12.3)	9.8 (9.0–10.3)	10.4 (9.5–12.4)	11.2 (10.0–12.3)	9.7 (8.2–10.8)	12.0 (11.0–12.6)
**Serology ANA, n (%)**						
Present	66 (22.4)	6 (27.3)	10 (19.2)	36 (24.0)	1 (14.3)	13 (20.3)
Absent	171 (58.0)	16 (72.7)	13 (25.0)	94 (62.7)	6 (85.7)	42 (65.6)
Not done	58 (19.7)	0 (0.0)	29 (55.8)	20 (13.3)	0 (0.0)	9 (14.1)
**Serology RF, n (%)**						
Present	23 (8.0)	0 (0.0)	4 (8.2)	13 (8.7)	1 (14.3)	5 (8.2)
Absent	195 (67.7)	22 (100.0)	15 (30.6)	112 (75.2)	5 (71.4)	41 (67.2)
Not done	70 (24.3)	0 (0.0)	30 (61.2)	24 (16.1)	1 (14.3)	15 (24.6)
**Serology HLA B27, n (%)**						
Present	14 (5.1)	0 (0.0)	2 (5.6)	8 (5.3)	0 (0.0)	4 (6.7)
Absent	103 (37.9)	10 (52.6)	2 (5.6)	63 (42.0)	2 (28.6)	26 (43.3)
Not done	155 (57.0)	9 (47.4)	32 (88.9)	79 (52.7)	5 (71.4)	30 (50.0)
**Serology anti CCP, n (%)**						
Present	11 (4.2)	0 (0.0)	3 (7.3)	6 (4.0)	1 (14.3)	1 (1.8)
Absent	72 (27.2)	6 (50.0)	5 (12.2)	26 (17.3)	2 (28.6)	33 (60.0)
Not Done	182 (68.7)	6 (50.0)	33 (80.5)	118 (78.7)	4 (57.1)	21 (38.2)
**CHAQ** (*n* = 28), Median (IQR)	1.3 (0.7–2.0)	1.3 (1.0–1.7)	6.0 (6.0–6.0)	NA (NA – NA)	NA (NA – NA)	0.2 (0.0–2.5)
**JADAS 10 score** (*n* = 105), Median (IQR)	10.3 (4.8–18.2)	13.0 (8.9–19.5)	26.0 (17.8–31.0)	3.0 (3.0–3.2)	6.0 (5.5–8.5)	10.2 (5.0–16.0)
**Medication at Diagnosis**						
bDMARD + NSAID	3 (1.7)	0 (0.0)	1 (4.0)	0 (0.0)	0 (0.0)	2 (3.5)
csDMARD only	32 (18.1)	10 (58.8)	4 (16.0)	12 (16.4)	0 (0.0)	6 (10.5)
csDMARD + bDMARD	5 (2.8)	2 (11.8)	1 (4.0)	1 (1.4)	0 (0.0)	1 (1.8)
csDMARD + bDMARD + NSAID	2 (1.1)	0 (0.0)	0 (0.0)	0 (0.0)	0 (0.0)	2 (3.5)
csDMARD + bDMARD + Steroid	4 (2.3)	1 (5.9)	0 (0.0)	0 (0.0)	0 (0.0)	3 (5.3)
csDMARD + NSAID	31 (17.5)	0 (0.0)	4 (16.0)	8 (11.0)	0 (0.0)	19 (33.3)
csDMARD + Steroid	9 (5.1)	3 (17.6)	3 (12.0)	2 (2.7)	0 (0.0)	1 (1.8)
csDMARD + Steroid + NSAID	9 (5.1)	0 (0.0)	0 (0.0)	9 (12.3)	0 (0.0)	0 (0.0)
NSAID only	55 (31.1)	0 (0.0)	6 (24.0)	29 (39.7)	0 (0.0)	20 (35.1)
Steroid only	17 (9.6)	0 (0.0)	4 (16.0)	8 (11.0)	5 (100.0)	0 (0.0)
Steroid + NSAID	6 (3.4)	0 (0.0)	1 (4.0)	4 (5.5)	0 (0.0)	1 (1.8)

^1^n (%); Median (IQR)

^2^Frequency (%)

ESR-Erythrocyte sedimentation rate

CRP-C reactive protein

Hb-Haemoglobin

ANA-Antinuclear Antibody

RF-Rheumatoid Factor

HLA B27-Human Leucocyte Antigen B27

Anti CCP-anti cyclic citrullinated peptide

csDMARD-Synthetic Disease Modifying Anti-Rheumatic Drug

bDMARD-Biologic Disease Modifying Anti-Rheumatic Drug

NSAID-Non-Steroidal Anti-Inflammatory Drug

NA – Missing data

**Observation of ‘other’ clinical features**

Libya:

1. Abdominal pain

2. Bloody diarrhea

3. Chronic abdominal pain with oral ulcer

4. Morning stiffness with abdominal pain and weight loss

5. Cushioned face due to oral steroid use

6. Fatigue

7. Weight loss

8. Headache

9. Mouth Ulcers

10. Scaly rash, bone pain, vitiligo

11. Raynauds’s phenomenon

Kenya:

1. Pyelitis with bloody diarrhea

2. Septic Arthritis

3. Withdrawn behavior suggesting depression

4. Recent upper respiratory tract infection

5. A week prior to onset had URTI and reported to have fallen in a bath

6. Hypopigmented rash with poor appetite

7. Loss of weight and appetite x2

8. Lethargy

## Discussion

We enrolled 302 patients diagnosed over a period of 23 years, 58.6% of whom were female. The JIA categories included 27.4% (83) oligoarthritis (OligoJIA), 17.9% (54) RF-polyarticular JIA (RF-pJIA), 5% (15) RF + polyarticular JIA (RF + pJIA), 7% (16) PsJIA, 18.2% (55) ERA, 18.9% (57) systemic JIA (SJIA), and 5.6% (17) undifferentiated arthritis. Our results are similar to the findings by Al Mayouf and colleagues in their study that found oligoarticular JIA to be the most predominant form of JIA in Africa and Middle East [13]. However, in our study, the countries in sub-sahara Africa had a higher proportion of systemic JIA and polyarticular JIA. This implies that there may be regional differences or the children with the more aggressive forms of the disease are the ones who seek care at these facilities. More data will be required to ascertain if these differences are significant. Due to under-representation of certain regions in Africa, a critical first step would be empowering the workforce in these regions to identify and manage JIA patients. This would help in generating data to inform practice. To substantiate the existence of a genuinely distinct subtype profile among African countries, further comprehensive epidemiologic data is imperative, given the current dearth of data in our continent.

The epidemiological data of enrolled patients in the registry is similar to various other research articles on JIA patients, particularly in terms of gender distribution and category prevalence [13–15]. However, our findings reveal a notable disparity among African countries concerning the age at inclusion and diagnosis delay. Despite Oligo-articular JIA being the most predominant category, the median age at diagnosis was 7 years yet the peak age for this category is 2–4 years [21]. In Kenya and Nigeria where polyarticular JIA was among the predominant categories, the median age was 9 and 12 years respectively yet biphasic peaks are reported globally of 1–4 years and 6–12 years [21]. Egypt exhibits a lower diagnosis delay compared to other countries. This observed distinction correlates with the high number of the rheumatology workforce in Egypt compared to other countries in Africa. This trend is similar to other studies across the globe [10–12, 17–38].

**Fig. 2:**
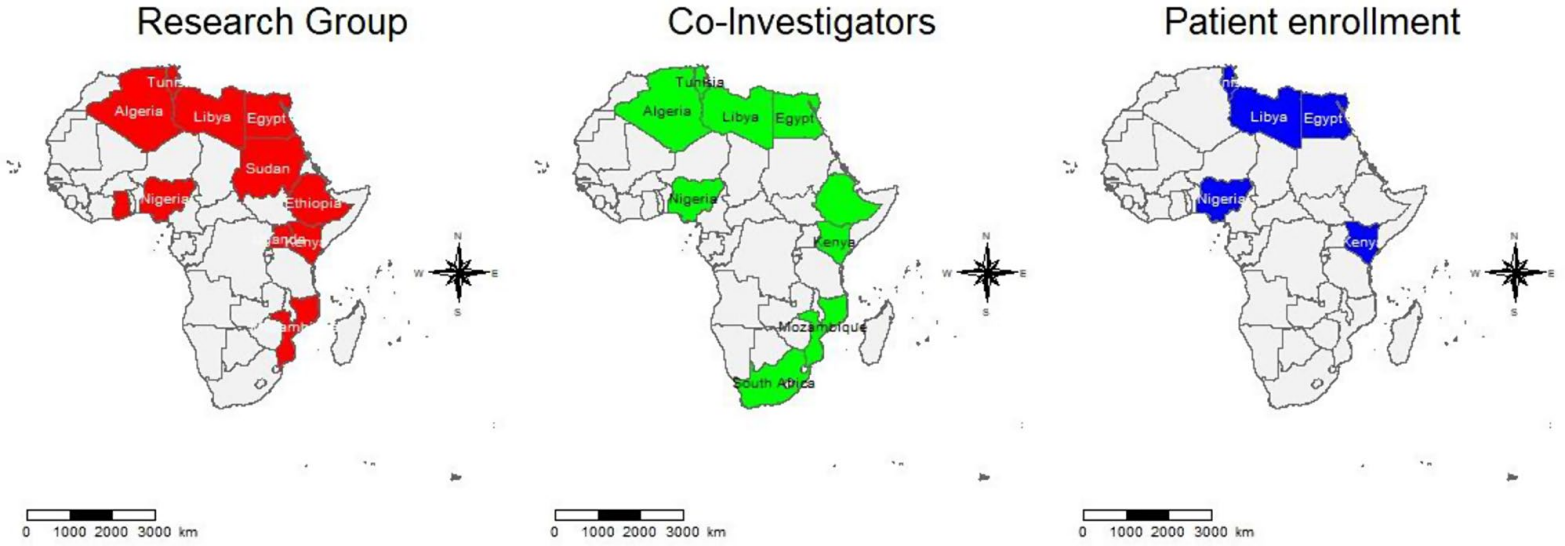
PAFLAR’s JIA registry activity in Africa. Red - Countries with PAFLAR research members contributing to the JIA Registry through subcommittees: Scientific, Ethics, Investigation Coordination, and Data Management & Manuscripts. Green - These African countries are either undergoing or have completed induction to become co-investigators in the registry. Those that have completed induction are awaiting ethical approvals before adding data. Blue - These five countries have obtained ethical approvals and have begun inputting data into the registry

Figure 2 illustrates the African member state countries that are members of the PAFLAR research working group and those that participated in our PAFLAR registry. This mirrors the countries with paediatric rheumatology centres as highlighted in one of our previous publications [8].

As regards treatment, the commonest therapies were NSAID therapy at 31.1%, synthetic DMARDs at 18.1%, synthetic DMARDs combined with NSAIDs at 17.5% and steroid therapy at 9.6%. Biological DMARDs accounted for 2.3% of therapies offered to our patients. The average JADAS score was 10.3 (range 3.8–17) and the average CHAQ score was 1.3 (range 0.65–1.95). Treatment options are influenced by the disease category and the medications available among other factors [39]. The predominant JIA category in our cohort was Oligoarticular JIA which might explain the predominantly high proportion of NSAID use. Nonetheless, the use of current synthetic and biological disease modifying antirheumatic drugs (DMARDs) requires more research on the optimal timing and duration applicable within our context [39]. Our findings reveal a limited overall utilization of biologics, accompanied by a considerable disparity in the usage patterns of steroids, NSAIDs, and csDMARDs within the studied cohort. This was highlighted in one of our previous publications that reveals the variation in availability of DMARD therapy and biological therapies [8]. This observed variation may be attributed to the economic conditions prevalent in African countries, further compounded by disparities in the distribution of categories within this demographic group. The discerned heterogeneity in medication utilization underscores the multifaceted nature of therapeutic decision-making processes within the context of the economic landscape and the diverse spectrum of JIA categories prevalent in this population.

In our cohort, we noted the lower frequency of uveitis compared to other international cohorts or registries. This was reiterated by Al Mayouf and co-authors who reported the incidence of uveitis and anti-nuclear antibody (ANA) positivity to be lower as compared to the incidence from other regions globally [13]. Addressing this disparity requires concerted efforts to implement routine screening for uveitis and enhance accessibility to ophthalmology care throughout our continent.

The absence of data pertaining to disease activity and functionality scores (CHAQ and JADAS) in certain countries is notable, as routine evaluation of these scores is not uniformly conducted across all centers. This emphasizes the imperative need for the implementation of strategic initiatives aimed at enhancing the capacity of healthcare professionals specializing in pediatric rheumatology throughout our continent to regularly assess and document the quality of life among JIA patients [40, 41]. The prospective segment of the PAFLAR registry, incorporating prospective follow-up data, is anticipated to address and rectify this existing gap.

As of the date of data extraction, the PAFLAR JIA registry had successfully enrolled 302 patients from eight centers across five African countries. However, this falls below our initial set target of enrolling all countries with paediatric rheumatology centres. We estimate we have 25 paediatric rheumatologists across Africa with Ethiopia being the most recent country to have its first paediatric rheumatologist. One of the challenges faced was the protracted and different requirements to obtain local ethical approval among various African member states. In addition, financial constraints made it difficult to compensate members for their time to input data hence we relied on their goodwill and willingness to support our registry. Some countries had strict data sharing laws which we postulate might have discouraged some members from enrolling patients into our registry. Another limitation we faced was that data collection was fragmented and partial hence limiting the epidemiological value of the study. Hence, among our 11 African member states only 5 participated in our PAFLAR JIA Registry. We hope to overcome some of these challenges by lobbying for more resources to support ethical approval applications and compensate members for their time and efforts in participation in our registry. We hope publication of this manuscript can help motivate other African member states to appreciate the importance of collective efforts in generating data and motivate them to participate in similar future initiatives. Nonetheless, our study provides insight on the profound heterogeneity of access to primary care and treatments in different countries across Africa.

We shall endeavor to continue with our social media and awareness campaign among our members to highlight the benefits of collaborative efforts and importance of participation in the PAFLAR JIA Registry. In view of this, we plan to administer a feedback survey amongst our research working group members to identify potential barriers affecting both patient and investigator recruitment. Our goal is to thoroughly understand these challenges and devise effective solutions to enhance enrollment in the PAFLAR JIA registry.

In order to overcome challenges in obtaining ethical approval, our ethics subcommittee actively engages with potential investigators, offering guidance and support to streamline the approval process with their respective local ethical committees. The aim is to navigate and address any variations or hurdles in ethical approval across different regions.

Recognizing the power of social media, we launched a dedicated campaign to raise awareness and attract potential participants. This initiative aims to tap into online platforms to disseminate information about the registry, creating a broader reach and engagement within the community.

Thus, to optimize recruitment we endeavour to implement a holistic approach combining multiple recruitment strategies and channels, tailoring communication with the native language of the target audience, being active (mobilize teams), reactive (provide prompt technical support), and proactive (share regular updates and reminders) [42]. Increasing awareness within the community about the existence of the registry and its potential outcomes could assist us in addressing these challenges [42].

## Conclusion

We successfully created the first Pan-African PAFLAR (JIA) registry in Africa and described the first large cohort of JIA patients in the continent. The PAFLAR JIA registry not only brings research practices to meet international standards but creates a platform to evaluate the clinical effectiveness and safety of various therapeutic interventions across diverse healthcare systems, taking into account specific epidemiological, infectious, and environmental factors. We hope data from this registry will contribute towards enhancing the overall quality of pediatric rheumatology care throughout the continent.

In addition, the registry serves as a foundation for progress in research, treatment methodologies, and community education. Its objective is to foster a more informed and supportive environment for individuals grappling with these chronic illnesses. The PAFLAR JIA registry is an ongoing initiative that welcomes participation from all African countries. This inclusive approach not only encourages collaboration but also ensures a comprehensive representation of the diverse healthcare landscapes within the continent.

### Electronic supplementary material

Below is the link to the electronic supplementary material.

Supplementary Material 1

## Data Availability

Data extraction files are available upon reasonable request.

## Abbreviations

AKUH: Aga Khan University Hospital
CI: Confidence Interval
CHAQ: Childhood Health Assessment Questionnaire
DMARD: Disease Modifying Antirheumatic Drug
ERA: Enthesitis Related Arthritis
GALS: Gait, arms, legs, and spine tool
HLA B27: Human Leucocyte Antigen B27
IQR: Interquartile range
JADAS: Juvenile Arthritis Disease Activity Score
JIA: Juvenile Idiopathic Arthritis
MSK: Musculoskeletal
N: Sample size
NSAID: Non-Steroidal Anti-inflammatory Drug
PAFLAR: Paediatric Society of the African League Against Rheumatism
pGALS: Paediatric gait, arms, legs, and spine tool
p: Prevalence
REDCap: Research Electronic Data Capture
RE: Rheumatoid Factor
ROC: Receiver operating curve
Sn: Expected sensitivity
UK: United Kingdom
W: Assumed 95% confidence width
WHO: World health organisation
