# Supplementary material for: Development of the paediatric society of the African league against rheumatism (PAFLAR) JIA registry and clinical profile of JIA in Africa from the PAFLAR JIA registry
Source: Pediatr Rheumatol Online J. 2024 Jul 22;22:67. doi: 10.1186/s12969-024-01000-3 (PMC11264440; doi:10.1186/s12969-024-01000-3)
Supplement: Supplementary file 1 — Supplementary Material 1 [file 12969_2024_1000_MOESM1_ESM.docx]

Supplementary Table

**Research centres with ethical approval and enrolment dates**

| **Institution** | **Country** | **Ethical approval from the local institution** | **Center Ethical approval reference number** | **PAFLAR center approval** | **PAFLAR Ethical approval reference number** | **Date of 1st patient inclusion** |
| --- | --- | --- | --- | --- | --- | --- |
| Tunis El Manar University  Kassab Institute | Tunisia | April 2022 | IMKO-CE-2022-001 | August 2022 | 022/PAFLAR-IERC-002 | 7/16/2022 |
| Tanta University | Egypt | May 2022 | 35470/5/22 | August 2022 | 2022/PAFLAR-IERC-001 | 6/15/2022 |
| Mansoura university | Egypt | June 2022 | R.22.05.1713 | August 2022 | 2022/PAFLAR-IERC-003 | 7/9/2022 |
| Gertrude’s Children’s Hospital | Kenya | August 2022 | GCH289/2022 | October 2022 | 2022/PAFLAR-IERC-004 | 8/29/2022 |
| Aga Khan university, Nairobi | Kenya | Sept 2022 | 2022/ISERC650  (v2) | October 2022 | 2022/PAFLAR-IERC-005 | 5/03/2022 |
| University of Tripoli | Libya | Dec 2022 | SREC-UOT 14-2022 | January 2023 | 2023/PAFLAR-IERC-007 | 11/2/2023 |
| Tunis El Manar University  Mongi Slim hospital | Tunisia | Feb 2023 | 02/CE/2023 | July 2023 | 2023/PAFLAR-IERC-008 | 10/18/2023 |
| University teaching hospital, IKEJA, LAGOS STATE | Nigeria | March 2023 | LREC/06/10/210007/23 | July 2023 | 2023/PAFLAR-IERC-009 | 9/22/2023 |
| CHU Beni messous hospital Issad Hassani | Algeria | May 2023 | 2022-A0340 | June 2023 | 2023/PAFLAR-IERC-010 | - |
